# SpaceOAR hydrogel spacer in interstitial brachytherapy for intrapelvic recurrent endometrial cancer

**DOI:** 10.1259/bjrcr.20210220

**Published:** 2022-03-09

**Authors:** Yoshiaki Takagawa, Jun Itami

**Affiliations:** 1 Department of Minimally Invasive Surgical and Medical Oncology, Fukushima Medical University, Fukushima, Japan; 2 Department of Radiation Oncology, National Cancer Center Hospital, Tokyo, Japan; 3 Department of Radiation Oncology, Shinmatsudo Central General Hospital, Chiba, Japan

## Abstract

**Objective:**

We report the use of SpaceOAR hydrogel spacer in interstitial brachytherapy (ISBT) for a patient with intrapelvic recurrent endometrial cancer (EC).

**Methods and materials:**

A 59-year-old female patient was diagnosed with intrapelvic recurrence of EC after a definitive surgery. Despite administration of adjuvant chemotherapy, the recurrent tumour in the right para-rectal fossa increased in size. Salvage radiotherapy, including external beam radiotherapy followed by ISBT boost, was planned. We planned to inject SpaceOAR between the tumour and rectum to reduce the rectal dose in ISBT; transrectal ultrasound-guided SpaceOAR injection was performed using needle applicator insertion. This was followed by computed tomography-based image-guided brachytherapy.

**Results:**

The use of SpaceOAR allowed us to achieved both a higher dose for the clinical target volume and a lower dose for the rectum. Furthermore, no ISBT-related complications or acute toxicities were observed.

**Conclusions:**

The preliminary results suggest that SpaceOAR could be effective in increasing the efficacy of ISBT for intrapelvic recurrent EC, while reducing the associated complications.

## Introduction

Endometrial cancer (EC) is the most common gynaecological malignancy in developed countries; it is the fourth leading cause of cancer and the sixth leading cause of cancer-related deaths among females in the United States.^1^ Post-operative intrapelvic recurrence of EC is often associated with the recurrent tumour being closely related to the small/large bowel and rectum. This makes it difficult to prescribe an adequate radiation dose to the recurrent tumour during salvage radiotherapy. Since 2015, the use of SpaceOAR hydrogel spacer (Boston Scientific, Marlborough, MA, USA) has played an important role in radiotherapy (RT) of prostate cancer.^2^ SpaceOAR has been demonstrated to attain a significantly lower incidence of rectal toxicity with a median follow-up period of 3 years.^3^ A similar rectal spacer for RT of gynaecological malignancies has not yet been developed.

Herein, we reported a patient with intrapelvic recurrent EC treated with external beam radiotherapy (EBRT) followed by an interstitial brachytherapy (ISBT) boost while using SpaceOAR to reduce the rectal dose.

## Clinical presentation

A 59-year-old female was diagnosed with endometrial clear cell carcinoma. Total abdominal hysterectomy, bilateral salpingo-oophorectomy and omentectomy were performed. Intraoperative findings revealed several nodules in the pouch of Douglas and omentum, and peritoneal dissemination was strongly suspected. Therefore, the peritoneal disseminated nodules were excised to as great an extent as possible. Although the surgery successfully resected all the gross tumours, lymph node dissection could not be performed because of intraoperative bleeding and the long operation time. Intraoperative cytology of the ascites was also positive (class V). The patient was diagnosed with T3aN0M1, pathological Stage IVB EC. Therefore, six cycles of docetaxel plus cisplatin as adjuvant systemic chemotherapy were administered.

## Imaging findings

**Figure 1.**
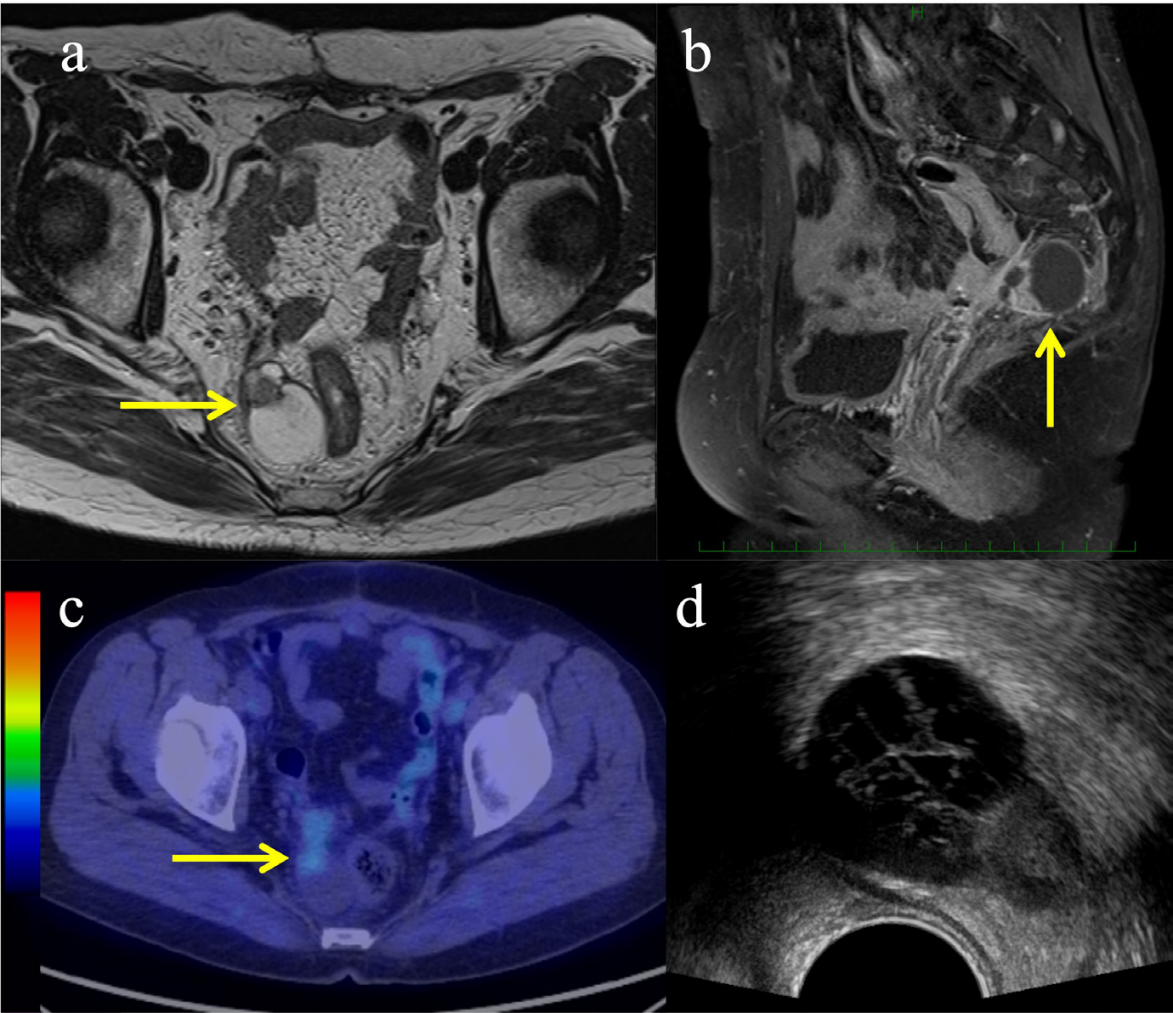
Pre-treatment images of the recurrent endometrial cancer. (a–b) Magnetic resonance imaging showing a tumour with a parenchymal and cystic component (yellow arrows): (**a**) Axial view of a *T*_2_-weighted image; (**b**) Sagittal view of a *T*_1_-weighted contrast-enhanced image; (**c**) Fluorodeoxyglucose uptake showing the parenchymal component of the tumour on positron emission tomography (yellow arrow); (**d**) Axial image of the transrectal ultrasound.

However, 5 months after surgery, a follow-up computed tomography (CT) scan revealed an enlarging tumour with a parenchymal and cystic component in the right para-rectal fossa. Although this cystic lesion had already been identified in the pre-surgery CT, it had been considered to be a benign cyst. Pelvic magnetic resonance imaging (MRI) at 6 months after surgery is shown in Figure 1a and b. Positron emission tomography (PET)-MRI revealed fluorodeoxyglucose (FDG) accumulation in the parenchymal component of the tumour (Figure 1c), and no other metastatic or recurrent lesions were found. The size of the recurrent tumour was 41 mm × 27 mm × 38 mm. We speculated that the tumour had persisted in the right para-rectal fossa and increased in size after chemotherapy. Therefore, the recurrent tumour was considered chemoresistant, and RT as local treatment was recommended.

## Treatment

Transrectal ultrasound (TRUS) was performed to evaluate whether ISBT could be considered. An axial view of TRUS of the tumour is shown in Figure 1d. The tumour was located in the right para-rectal fossa, close to the rectum. Therefore, we considered that TRUS-guided ISBT with percutaneous needle application was possible. To reduce the rectal dose in ISBT, we planned to inject SpaceOAR between the tumour and the rectum. SpaceOAR is officially permitted only for prostate cancer in national health insurance. Hence, for the use of the SpaceOAR in the current case, we obtained informed consent from the patient and an approval of the institutional review board for extrainsurance usage of SpaceOAR for EC. EBRT followed by a high-dose rate (HDR) ISBT boost was planned. The EBRT technique that was used for the whole pelvis was intensity-modulated RT. The EBRT dose was 50 Gy in 25 fractions.

## Outcome, follow-up and discussion

**Figure 2.**
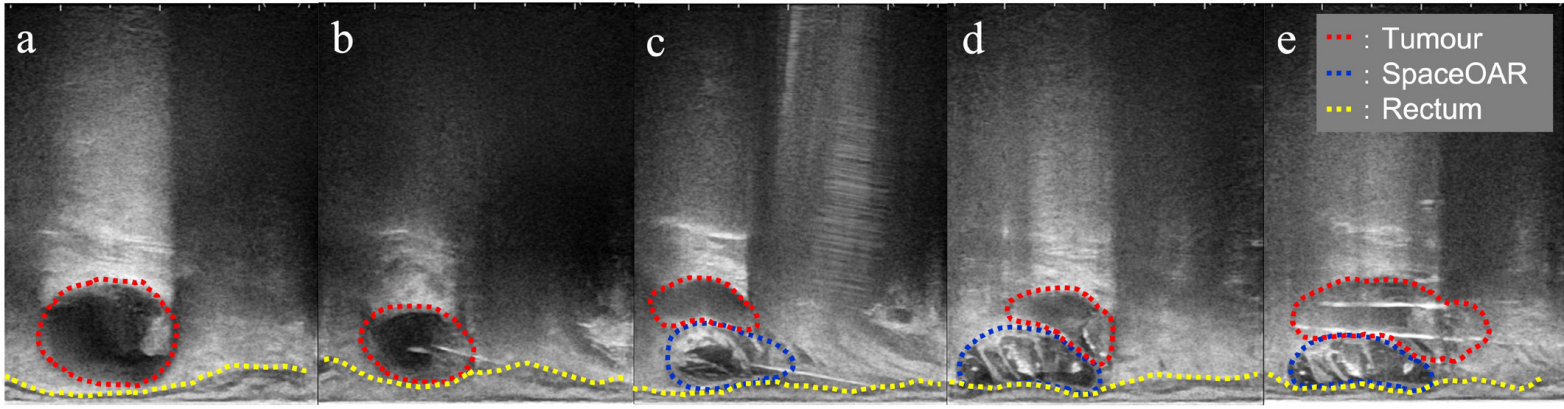
Sagittal view of the transrectal ultrasound of the procedure of the SpaceOAR hydrogel spacer injection and interstitial needle insertion. (**a**) The recurrent tumour with a parenchymal and cystic component (red dotted line; tumour, yellow-dotted line; rectum); (**b**) Needle puncture of the cystic component of the tumour; (**c**) SpaceOAR was injected between the tumour and rectum (blue-dotted line; SpaceOAR); (**d**) After injection of the SpaceOAR; (**e**) Placement of the interstitial needles for the tumour.

**Figure 3.**
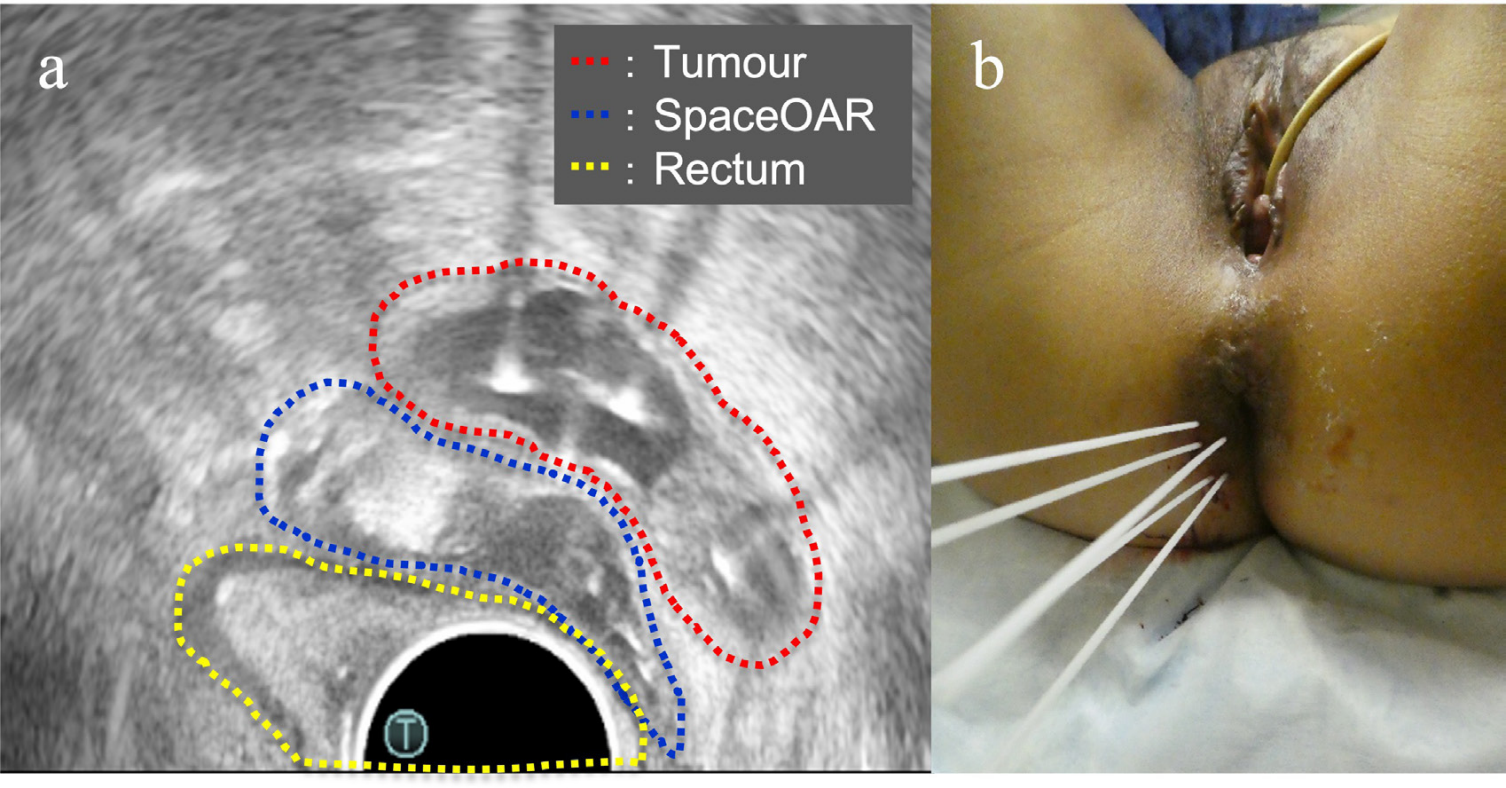
(**a**) Axial view of the transrectal ultrasound after implantation of the SpaceOAR hydrogel spacer and interstitial needles. This shows that the SpaceOAR is placed in an optimal position between the tumour and rectum; (**b**) Image of the interstitial brachytherapy.

**Figure 4.**
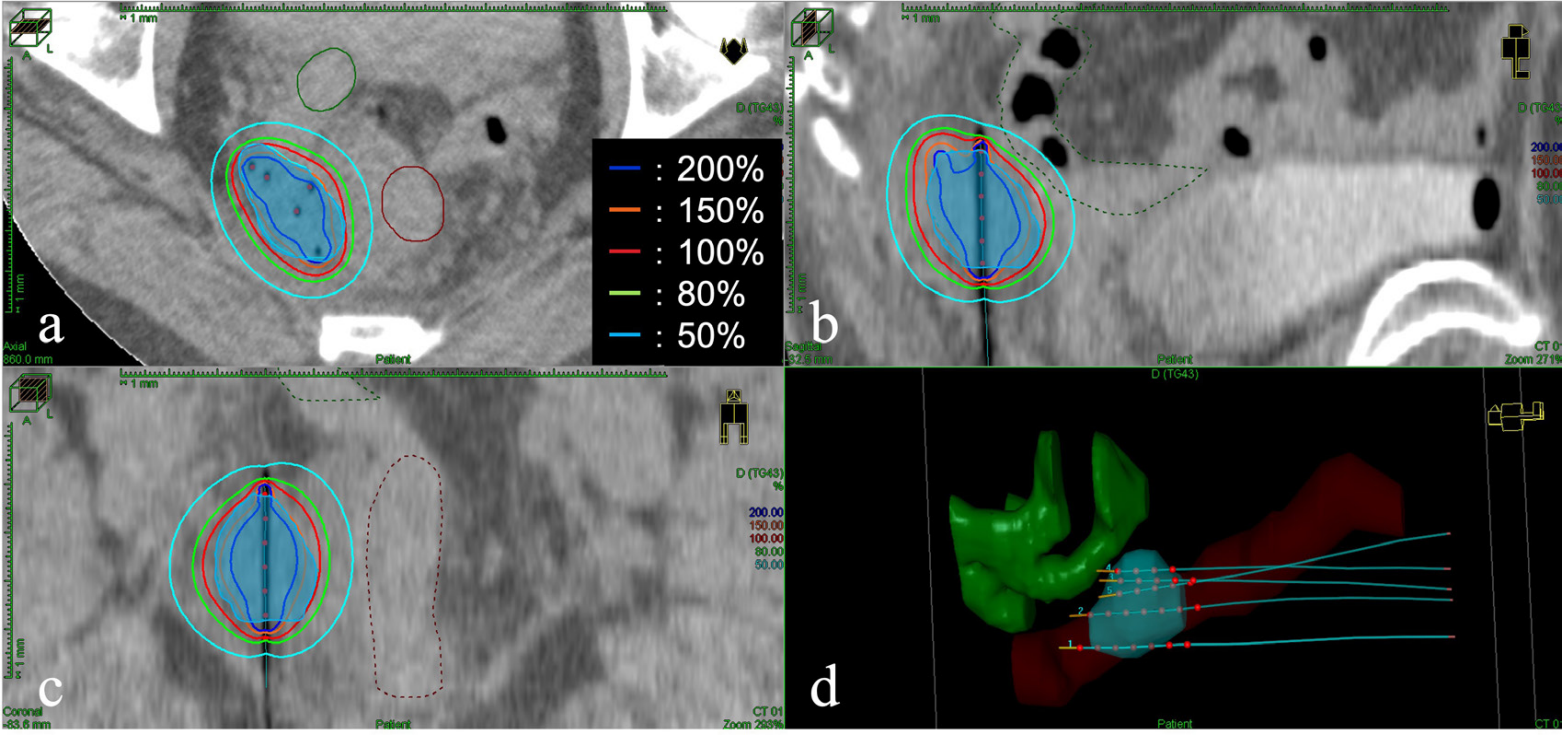
The image of dose distribution of interstitial brachytherapy. (**a**) Axial view; (**b**) Sagittal view; (**c**) Coronal view; (**d**) Three-dimensional reconstruction of the target, organ at risk, and five interstitial needles. Light blue shading: clinical target volume; brown lines and shading: the rectum; green lines and shading: the small bowel. The red line is the 100% (6 Gy) isodose line.

**Figure 5.**
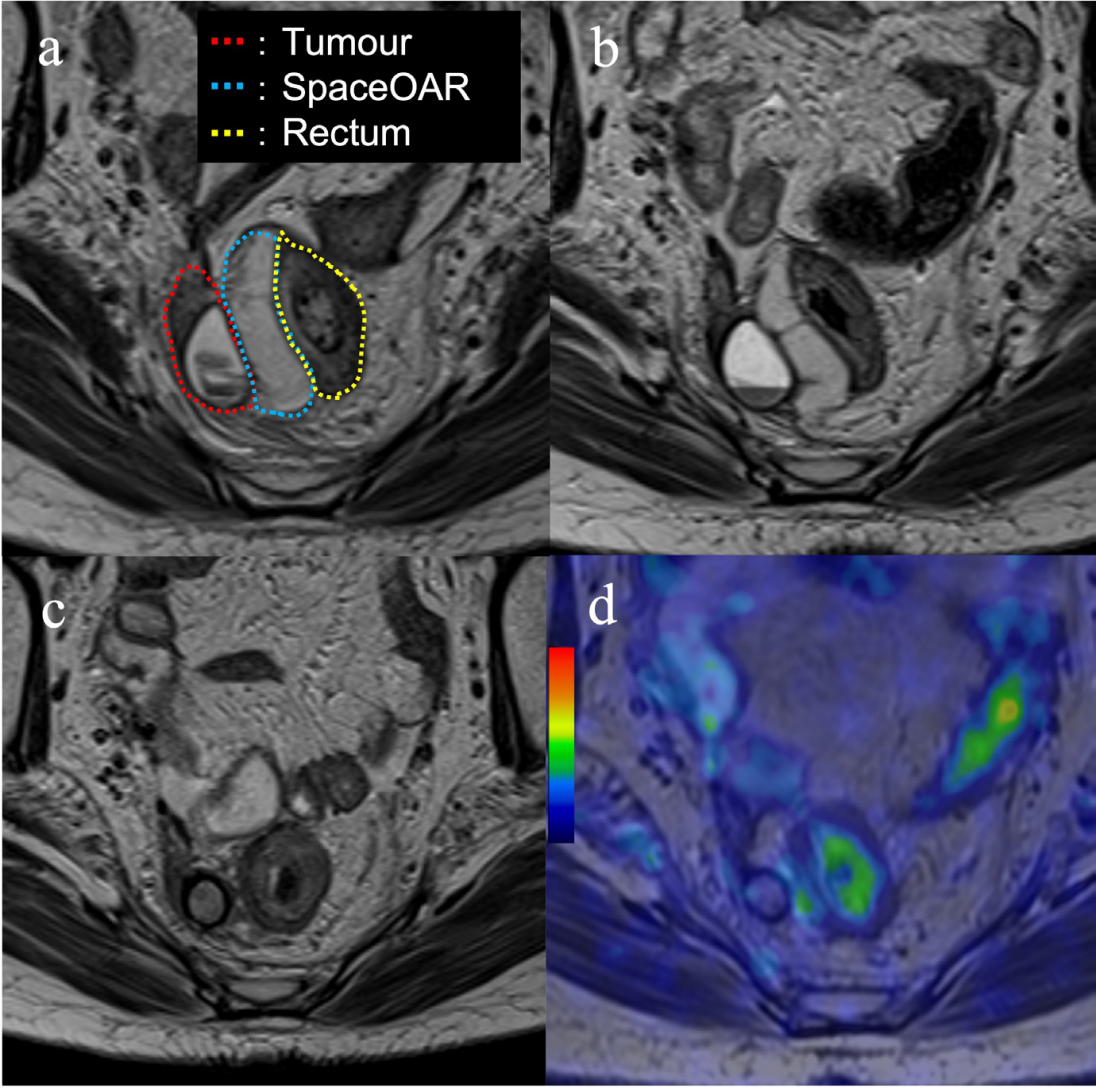
Images of the magnetic resonance imaging during and after treatment. (**a**) Four days after the first interstitial brachytherapy (red dotted line; tumour, blue dotted line; SpaceOAR, yellow dotted line; rectum); (**b**) One month after treatment; (**c**) Four months after treatment. The SpaceOAR is absorbed completely and has disappeared; (**d**) Image of the positron emission tomography-magnetic resonance imaging 8 months after treatment. There is no fluorodeoxyglucose accumulation in the recurrent tumour.

Post completion of EBRT, MRI revealed no significant change in the tumour, and ISBT was performed weekly. We performed CT-based image-guided brachytherapy (BT). ProGuide sharp needle applicators of 5 Fr (Elekta, Sweden) were used under TRUS guidance. An intravenous injection of pentazocine, hydroxyzine hydrochloride, and midazolam was administered for anaesthesia during applicator insertion. The procedure for ISBT under TRUS guidance is shown in Figure 2. In the first ISBT, we performed a needle puncture for the cystic component of the tumour to reduce tumour volume before SpaceOAR and interstitial needle insertion (Figure 2a and b). The punctured fluid was cytologically positive for malignancy (class IV). Next, we injected SpaceOAR between the tumour and the rectum (Figure 2c and d). We inserted SpaceOAR injection needle to aim at the space between the tumour and the rectum so as not to damage the rectum wall. Then, we injected the 10 cc SpaceOAR between the rectum and the tumour following the same procedure used in patients with prostate cancer. Only one SpaceOAR injection was administered. After that, five interstitial needles were inserted into the recurrent tumour through its parenchymal and cystic components (Figure 2E). The axial TRUS image with SpaceOAR and the applicators *in situ* and photographs of ISBT are shown in Figure 3. The SpaceOAR injection procedure and every planning CT scan were performed in lithotomy position. The planning CT slice interval was 4 mm. For HDR-BT, we used Ir-192 with a microSelectron^®^ Digital (HDR-V3) Brachytherapy Afterloader and the Oncentra^®^ Brachy treatment planning system (Elekta, Sweden). The BT dose was 24 Gy delivered in four fractions to the recurrent tumour as the clinical target volume (CTV). We aimed for a 100% prescription isodose line to fit the CTV. The dose distribution of ISBT is shown in Figure 4. Due to the SpaceOAR, we achieved both a higher dose for the CTV and a lower dose for the rectum. Four days after the first ISBT, MRI revealed that SpaceOAR was placed between the tumour and rectum precisely, and that it was in perfect correspondence with the real-time TRUS image (Figures 3a and 5a). In the third ISBT, the cystic component of the tumour increased in size. Therefore, before needle insertion, we again performed needle puncture for the cystic component of the tumour to reduce the tumour volume. The punctured fluid was diagnosed as having no malignant cells on cytology (class II). The interstitial needles were inserted and removed with each ISBT procedure. Therefore, the needle position and the dose distribution were optimised for each ISBT. The dosimetric parameters of each ISBT are listed in Table 1. The average CTV D90 and V100 values were 7.9 Gy and 99.4%, respectively. The total biologically equivalent dose in 2 Gy fractions (Gy_EQD2_) of EBRT (50 Gy/25 fractions) plus ISBT (24 Gy/4 fractions), based on the linear-quadratic model for CTV D90, was 97.3 Gy_EQD2_, assuming an α/β ratio of 10. For Gy_EQD2_ of the organs at risk (OAR), an α/β ratio of 3 Gy was assumed. The total Gy_EQD2_ of EBRT plus ISBT for the rectum and small bowel were 58.5 Gy_EQD2_ and 56.4 Gy_EQD2_. There were no ISBT-related complications or acute toxicities.

**Table 1.** Dosimetric parameters of each brachytherapy

Brachytherapy	CTV D90 (Gy)	CTV V100 (%)	Rectum D2cc (Gy)	Small bowel D2cc (Gy)
# 1	7.8	99.68	2.7	1.9
# 2	8.0	99.83	1.4	1.9
# 3	7.6	98.57	1.6	1.5
# 4	8.3	99.48	2.5	1.5

CTV, clinical target volume; D_90_, minimal dose delivered to 90% of target volume; V_100_, fractional volume of the organ receiving 100% of the prescribed dose; Rectum D_2cc_ and Small bowel D_2cc_, doses for most exposed 2 cc volumes of rectum and small bowel, respectively;

One month after ISBT, the recurrent tumour decreased in size, and the SpaceOAR remained (Figure 5b). Four months after ISBT, pelvic MRI revealed that the recurrent tumour, including both parenchymal and cystic components, had shrunk further, and the SpaceOAR was absorbed completely (Figure 5c). Eight months after ISBT, PET-MRI revealed that the recurrent tumour was of the size 10× 9 mm×11 mm, with no FDG accumulation detected (Figure 5d). However, para-aortic lymph node metastasis, which was outside the radiation field, and intrapelvic nodules suspected of peritoneal dissemination in the radiation field were revealed on PET-MRI. Subsequently, the patient received another round of systemic chemotherapy. The patient died of systemic progression of EC at 21 months after the ISBT. No tumour regrowth underwent BT during follow-up and no late recto-gastrointestinal toxicities of EBRT and ISBT were identified prior to the patient’s death.

We demonstrated that ISBT boost for intrapelvic recurrent EC using TRUS-guided SpaceOAR placement achieved excellent local control without causing significant morbidities. SpaceOAR is generally used only for patients with prostate cancer. However, the development of a spacer for gynaecological BT is strongly desired, and only few reports have been published on the use of spacers in gynaecological BT. In 2012, Marnitz et al^4^ published the first report of using SpaceOAR for rectal separation in five patients with cervical cancer treated with primary chemoradiotherapy. More recently, several spacers for gynaecological malignancies other than SpaceOAR have been under development. Basu et al^5^ reported the use of hydroxypropyl methylcellulose (Viscomet^®^) in intracavitary BT for cervical cancer. Damato et al^6^ performed a proof of concept study in a cadaveric model using TraceIT (Boston Scientific, Marlborough, MA, USA) as a spacer to separate the rectum, cervix and bladder. Ahmed et al^7^ reported two clinical cases of gynaecological BT with interventional radiological TraceIT injections. Kashihara et al^8^ and Murakami et al^9^ reported hyaluronic gel injection for rectum and bladder dose reduction in gynaecological BT. It is evident from all these reports, including our report, that the use of a spacer can attain a higher dose administered to the target without increasing the doses administered to the OARs and without significant toxicity. In the present case, because the recurrent tumour was closely related to the rectum, it was not possible to administer a sufficient dose to the target without using a spacer. Although the placement of these spacers requires technical expertise, if the spacers are placed in the appropriate site, it can provide a clinically significant benefit for the patient. As shown in Figures 3a and 5a, SpaceOAR was placed into the appropriate site using TRUS-guided injection. Using SpaceOAR, we were able to significantly lower the rectal dose (Table 1), and as a result, we were able to administer a larger dose to the target. SpaceOAR Hydrogel stays in place for about 3 months and is naturally absorbed into the body and removed through urine within 6 months (Figure 5c and d). Therefore, appropriately placed spacers can not only lower acute/late toxicity but also attain a higher local control rate. This case shows that short and middle-term tumour control can be safely achieved in patients with gynaecological malignancy treated with interstitial brachytherapy using SpaceOAR. To the best of our knowledge, this is the first report of ISBT for a patient with intrapelvic recurrent EC using SpaceOAR injection for rectal dose reduction.

Our study has some limitations. There is a possibility of peritoneal dissemination caused by ISBT. In the case presented here, the recurrent tumour had both parenchymal and cystic components. We performed needle puncture for the cystic component of the tumour to reduce the tumour volume and penetrated the interstitial needle applicators through the cystic component. However, the patient had already been diagnosed with peritoneal nodules and positive ascites cytology at the initial surgery; therefore, we considered that our ISBT procedure did not influence peritoneal recurrence after ISBT. Second, the tissue status of patients with the intrapelvic recurrent gynaecological malignancies may differ significantly from that of patients with prostate cancer. Therefore, for patients with gynaecological cancer, clinicians should consider whether using the SpaceOAR system would provide an adequate space between the target and OAR on a case-by-case basis.

ISBT using a SpaceOAR hydrogel spacer for intrapelvic recurrent EC is effective and safe. If a safe approach to place the spacer is achieved, selected patients may have a large benefit from BT. Further studies on the use of spacer in RT for gynaecological malignancies are required to confirm this.

## Learning points

- ISBT using a SpaceOAR hydrogel spacer for intrapelvic recurrent EC is effective and safe.
- The use of SpaceOAR achieves both a higher dose to the clinical target volume and a lower dose to the rectum.
